# Evaluation of Influenza Prevention in the Workplace Using a Personally Controlled Health Record: Randomized Controlled Trial

**DOI:** 10.2196/jmir.984

**Published:** 2008-03-14

**Authors:** Florence T Bourgeois, William W Simons, Karen Olson, John S Brownstein, Kenneth D Mandl

**Affiliations:** ^1^Division of Emergency MedicineChildren’s Hospital BostonBostonMAUSA; ^2^Department of PediatricsHarvard Medical SchoolBostonMAUSA; ^3^Children’s Hospital Informatics Program at the Harvard-MIT Division of Health Sciences and TechnologyBostonMAUSA

**Keywords:** Randomized controlled trial, personally controlled health record, Web-based, employee health program, influenza

## Abstract

**Background:**

Personally controlled health records (PCHRs) are accessible over the Internet and allow individuals to maintain and manage a secure copy of their medical data. These records provide a new opportunity to provide customized health recommendations to individuals based on their record content. Health promotion programs using PCHRs can potentially be used in a variety of settings and target a large range of health issues.

**Objectives:**

The aim was to assess the value of a PCHR in an employee health promotion program for improving knowledge, beliefs, and behavior around influenza prevention.

**Methods:**

We evaluated a PCHR-based employee health promotion program using a randomized controlled trial design. Employees at Hewlett Packard work sites who reported reliable Internet access and email use at least once every 2 days were recruited for participation. PCHRs were provided to all participants for survey administration, and tailored, targeted health messages on influenza illness and prevention were delivered to participants in the intervention group. Participants in the control group received messages addressing cardiovascular health and sun protection. The main outcome measure was improvement in knowledge, beliefs, and behavior around influenza prevention. Secondary outcomes were influenza vaccine rates among household members, the impact of cardiovascular health and sun protection messages on the control group, and the usability and utility of the PCHR-based program for employees.

**Results:**

The intervention did not have a statistically significant effect on the influenza knowledge elements we assessed but did impact certain beliefs surrounding influenza. Participants in the intervention group were more likely to believe that the influenza vaccine was effective (OR = 5.6; 95% CI = 1.7-18.5), that there were actions they could take to prevent the flu (OR = 3.2; 95% CI = 1.1-9.2), and that the influenza vaccine was unlikely to cause a severe reaction (OR = 4.4; 95% CI = 1.3-15.3). Immunization rates did not differ between the intervention and control groups. However, participants in the intervention group were more likely to stay home during an infectious respiratory illness compared with participants in the control group (39% [16/41] vs 14% [5/35], respectively; *P* = .02). The program also succeeded in improving recognition of the signs of heart attack and stroke among participants in the control group. Overall, 78% of participants rated the PCHR as “extremely/very” easy to use, and 73% responded that they would be “extremely/very” likely to participate again in a PCHR-based health promotion system such as this one.

**Conclusions:**

With a small sample size, this study identified a modest impact of a PCHR-based employee health program on influenza prevention and control. Employees found the PCHR acceptable and easy to use, suggesting that it should be explored as a common medium for health promotion in the workplace.

**Trial Registration:**

ClinicalTrials.gov NCT00142077

## Introduction

Yearly influenza outbreaks are a prime example of a public health problem with well-developed surveillance methods and evidence-based programs for prevention but poor compliance with health protection guidelines [1]. Fifty-five million adults aged 18 to 64 years are infected with influenza every year, with 200 million days of restricted activity, 70 million days of work absenteeism, and 18 million visits to health care providers [2]. Influenza vaccination among healthy, working adults has been shown to be highly effective, resulting in a 25% reduction in any episode of upper respiratory illness, a 43% decrease in days of work missed due to respiratory illness, and 44% fewer visits to physicians’ offices for respiratory illnesses when compared to unvaccinated adults [3]. Nonetheless, vaccination rates are only 18% among healthy adults 18 to 49 years of age and 46% among those with high-risk conditions 50-64 years of age [4].

Personally controlled health records (PCHRs) [5] are a subset of personal health records [1,6] and enable an individual to assemble, maintain, and manage a secure copy of his or her medical data [7]. PCHRs are designed based on the principle that patients have the right to own and manage copies of their own medical histories, and they provide a virtual medical home with modalities for communication among patients, clinicians, and health authorities. PCHRs present a new opportunity to bridge the gap between public health research and action to improve the health of individuals. We explored the use of a PCHR as a vehicle for the delivery of customized health promotion messages in which individuals received information and recommendations based on their record content. This approach to health communication enables rapid, tailored, and targeted delivery of health care recommendations to individuals. Tailored communication has previously been shown to be superior to generic, population-based recommendations in achieving patient compliance [8] and can easily be implemented with PCHRs.

We report an evaluation of a PCHR-based employee health promotion program using a randomized controlled trial design. The principle objective was to assess the use of the PCHR to improve knowledge, beliefs, and behavior surrounding influenza prevention. There were three secondary objectives. The first was to assess the effect of electronic messages delivered through the PCHR on influenza vaccine rates among household members, the second was to assess the impact of messages addressing cardiovascular health and sun protection on the knowledge and behavior among participants in the control group, and the third was to evaluate the usability and utility of the PCHR-based program for employees.

## Methods

### Design and Participants

Using a randomized controlled trial design (ClinicalTrials.gov: NCT00142077), we evaluated an electronic PCHR system to modify knowledge, beliefs, and behavior around influenza. Participants were recruited from eight Hewlett Packard Corporation work sites in the northeastern United States in the fall of 2005. Employees at the research sites were recruited with two emails sent to their work email address by the company’s human resources department. The emails contained study information and invited potential participants to complete a brief set of questions to assess eligibility. Eligible volunteers were 18 years of age or older, comfortable reading and writing in English, part-time or full-time employees of the company and had reliable Internet access at work, school, or home and used email at least once every 2 days. In addition, participants could not have a history of a severe reaction to influenza vaccine or severe allergy to chicken eggs, since both of these conditions contraindicate use of the influenza vaccine. Enrollment was initially planned for October 2005; however, just prior to the original recruitment period, several Hewlett Packard work sites were closed and employees were relocated or laid off at several other sites. Therefore, the study began in November 2005 and our recruitment pool was smaller than anticipated. All participants electronically provided informed consent prior to study initiation. The study was approved by the Committee on Clinical Investigation at Children’s Hospital Boston, Boston, MA, USA.

Assignment to the intervention and control groups was performed at the level of the corporation work site in order to prevent employees at the same site from sharing information about the trial, including recommendations provided in the health messages. Prior to study initiation, we created two groups with four sites in each such that the number of employees in each arm was evenly distributed. The two groups were then randomly assigned to the intervention and control arms by a person unfamiliar with the details of the work sites. Participants in the study were informed that the study was to evaluate health promotion using a PCHR with an electronic messaging system and were masked as to whether they were in the intervention or control groups.

### Interventions

We used a PCHR system called PING [9-11] (new versions are called Indivo [12]), which is built to open standards on a flexible XML data model and is accessible over the Web. PING is designed to enable patients to own complete, secure copies of their medical record and to integrate information over time and across sites of care [5,11]. In this investigation, we tested the survey, decision support, and health messaging features of PING, and records did not include any health information beyond the data provided by subjects for this study. Enrolled subjects completed online health risk assessment surveys, the responses to which drove the decision support system to generate and send tailored health messages for participants in the intervention group. These messages were sent to participants’ PING record inbox, and participants were simultaneously notified with a standard, plain-text email instructing them to visit and log on to their PING record to review the message (Multimedia Appendix 1: PING Record Welcome Screen and Inbox).

### Data Collection for PCHR

Participants in the intervention and control groups completed three types of survey. The first was a baseline survey that was posted in their PING record immediately after registration was completed (Multimedia Appendix 2: Enrollment Survey). This survey collected demographic data; information on medical history; health-related behaviors; influenza risk factors; knowledge, beliefs, and behavior around influenza; and information related to Internet use. Information was also collected on household members, including their age; gender; attendance at work, school, or daycare; and behaviors and risk factors related to influenza. The baseline survey administered to the control group contained additional questions addressing routine health and knowledge and behaviors regarding cardiovascular health and sun protection.

The second survey was a biweekly survey consisting of a brief set of questions that was administered approximately every 2 weeks (Multimedia Appendix 3: Biweekly Survey). A total of seven of these surveys were administered between December 1, 2005, and March 1, 2006. Information was collected on recent respiratory illnesses in participants and household members, including duration of symptoms, missed work or school days, medication use, and health care utilization, and an update was obtained on their influenza vaccine status. Biweekly surveys for the control group included additional questions on routine health care use and recent gastrointestinal or other illness.

The third survey was an exit survey administered at the end of the study, 2 weeks after the last biweekly survey. It contained the same questions on influenza knowledge, beliefs, and behavior as the baseline survey, as well as questions to evaluate the electronic interface of the application, its usability, the content of the questions, and the overall utility of the PCHR-based program to participants. The survey administered to the control group additionally contained the same questions on knowledge and behaviors regarding cardiovascular health and sun protection administered in the baseline survey.

### Health Messages

Participants in the intervention group received different types of influenza-related health messages throughout the study period. Some of these were personalized based on the information provided in the baseline and biweekly surveys and were posted in the record after a participant completed one of these surveys. Messages were tailored to include advice for all household members, to identify individuals at high risk for influenza-related complications, and to provide information on respiratory illnesses if a participant or household member became ill with a respiratory infection. The health messages were also tailored based on the home addresses of participants to advise them of influenza activity in their area. Other messages contained general information and were provided on a weekly or monthly basis. The content of the health messages was regularly monitored throughout the study period to ensure that proper messages were being generated and transmitted to participants.

**Figure 1:**
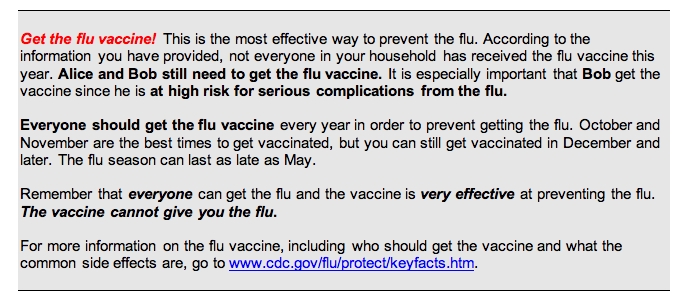
Sample health message: vaccine reminder (In this example, Alice and Bob are household members of the study participant.)

**Figure 2:**
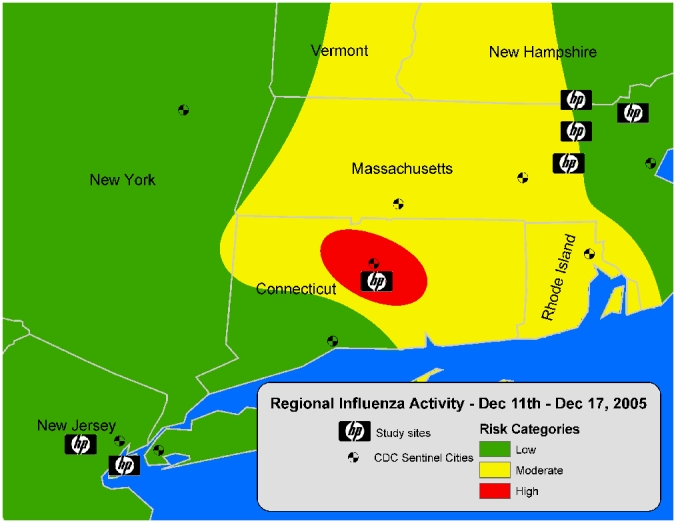
Sample health message: weekly influenza risk map (Risk categories were derived from data provided by the CDC on weekly mortality from pneumonia and influenza [9].)

There were five types of health message:

1. Vaccine reminders: If participants indicated that they or a household member eligible for the influenza vaccine were not yet vaccinated, a message was generated urging them to receive the vaccine. The message contained basic information on the influenza vaccine and identified any household members who were at high risk for influenza-related complications or severe disease based on recommendations by the Advisory Committee on Immunization Practices (ACIP) [13]. Figure 1 provides an example of such a personalized health message.
2. Respiratory illness advice: Information that a participant or household member had recently contracted a respiratory illness prompted a health message with advice on the treatment and prevention of respiratory illnesses (Multimedia Appendix 4: Sample Health Message). Participants were encouraged to stay home from work when ill, and guidelines were provided on when to contact a physician.
3. Influenza alerts: Based on surveillance information provided by the Centers for Disease Control and Prevention (CDC) on mortality related to influenza and pneumonia [14], weekly messages were sent to participants residing in areas with increased rates of death attributable to pneumonia and influenza. These messages alerted participants to the increase in influenza activity in their area and contained information on preventing influenza transmission.
4. Weekly influenza risk maps: Every week, participants received a map displaying areas of low, moderate, and high influenza activity in the northeastern United States. These maps were based on the weekly CDC surveillance of pneumonia and influenza [14] and kept participants informed of the spread of influenza in their region. Figure 2 is an example of such a map.
5. Monthly bulletins: Once a month, a message was sent with educational information about different aspects of influenza. A total of four such messages were sent, describing methods of influenza transmission and prevention, symptoms of influenza illness, influenza vaccine and its risks, and treatment options for influenza illness.

Participants in the control group received a monthly bulletin on cardiovascular health and sun protection (Multimedia Appendix 5: Control Group Monthly Bulletin). Information was selected based on Healthy People 2010 [15] objectives, which aim to reduce high-risk behaviors and improve the use of preventive services. Participants in the control group received neither personalized health messages nor information on influenza. Four bulletins were sent and provided information on cardiovascular disease, stroke, skin cancer and sun protection, and guidelines for a healthy diet.

### Outcomes

The primary outcome was change in knowledge, beliefs, and behavior surrounding influenza prevention. Change in knowledge was assessed using a set of nine questions in the baseline and exit surveys addressing influenza transmission and prevention, influenza illness, and the influenza vaccine. Change in beliefs was measured with a set of six questions on influenza illness and vaccine, administered in the baseline and exit surveys. Measurements of behavior change consisted of the rate of influenza vaccination, the rate of work attendance despite a respiratory illness, and responses to two questions in the baseline and exit surveys on hand hygiene and cough etiquette.

Secondary outcomes included the rate of influenza vaccination among household members and changes in knowledge and behavior regarding cardiovascular health and sun protection, measured using nine questions in the baseline and exit surveys administered to the control group. Finally, the usability and utility of the PCHR-based program were assessed by means of 12 questions in the exit survey, as well as survey completion rates and mean days to survey completion.

### Statistical Methods

Logistic regression models were used to analyze the changes in responses for the questions on knowledge and behavior surrounding influenza. The models controlled for baseline responses in the initial survey. A variable for participant work sites was tested in the models to control for clustering. This variable was not significant in any of the analyses and was excluded from the final models. Immunization rates were compared using chi-square analysis. Rates for missed work during an illness were examined using the SAS v9.1 (SAS Institute, Inc, Cary, NC, USA) PROC GENMOD procedure in order to control for correlated responses from participants with more than one illness. The knowledge and behavior questions on cardiovascular risk and sun protection for the control group were analyzed with the McNemar test. Assessment of the usability and utility of the program was performed through examination of the responses to the questions on user experience and calculation of completion rates and mean days to survey completion.

## Results

### Participation and Retention

**Figure 3:**
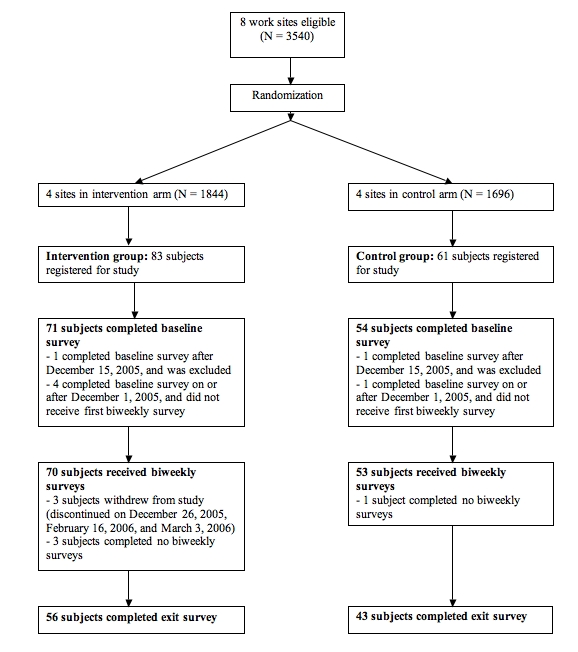
Flow diagram of study participation

Participant flow is shown in Figure 3. We recruited participants during a 4-week period between November 10 and December 7, 2005. Of the 3540 employees at the eight work sites, 144 employees registered for the study and 125 completed the baseline survey. Of these, 119 (95%) completed between one and seven biweekly surveys, and 99 (79%) completed the exit survey. The baseline characteristics of the intervention and control groups are shown in Table 1. Only the gender distribution differed between the two groups.

**Table 1:** Baseline characteristics of participants

Characteristic	Intervention (N = 71)	Control (N = 54)	*P* Value
Number of female participants (%)	41 (58)	20 (37)	.02
Mean age in years (SD)	46.4 (8.6)	46.9 (9.4)^*^	.47
Number at increased risk of complications^†^ (%)	10 (14)	9 (17)	.69
Number who received influenza vaccine during previous flu season (%)	19 (27)	13 (24)	.73
Number who received influenza vaccine during current flu season prior to study start (%)	14 (20)	9 (17)	.66

^*^One control subject excluded due to incorrect input of birth date.

^†^Based on recommendations by the Advisory Committee on Immunization Practices (ACIP) [13].

Of the 125 participants completing the baseline survey, two were excluded because they completed it too late (on March 16, 2006, and April 16, 2006). A total of 99 participants completed the exit survey and were included in the analyses examining changes in knowledge, beliefs, and behaviors. There were 123 participants who received biweekly surveys, among which four did not complete any of the biweekly surveys (or the exit survey) and were excluded from the analyses of vaccination rates and work attendance rates while ill. Among the control group, there were 43 participants who completed both the baseline and exit survey and were included in the analysis examining changes in knowledge and behavior regarding cardiovascular risk and sun protection. For the assessment of the usability and utility of the PCHR, we analyzed the responses to the questions on user experience in the exit survey completed by 99 participants, as well as the completion rates and times to completion of 123 participants for the enrollment survey, 119 participants for the biweekly surveys, and 99 participants for the exit survey.

### Outcomes and Estimation

#### Improvement in Knowledge, Beliefs, and Behavior Surrounding Influenza

Table 2 summarizes responses to survey questions evaluating knowledge, beliefs, and behaviors surrounding influenza. The intervention did not have a statistically significant effect on the knowledge elements we assessed. However, it did have a significant effect on certain beliefs surrounding influenza. At the end of the study, participants in the intervention group were more likely to believe that the influenza vaccine was effective (OR = 5.6; 95% CI = 1.7-18.5), that there were actions they could take to prevent the flu (OR = 3.2; 95% CI = 1.1-9.2), and that the influenza vaccine was unlikely to cause a severe reaction (OR = 4.4; 95% CI = 1.3-15.3). The intervention was not demonstrably effective in changing people’s beliefs that they should be immunized, that influenza illness is a moderately to extremely serious illness, or that immunization can help prevent influenza in other people. The two questions addressing hand hygiene and cough etiquette did not show any changes in behavior among the intervention group.

**Table 2:** Effect of intervention on knowledge, beliefs, and behavior regarding influenza

	Intervention (N = 56)		Control (N = 43)		OR (95% CI)^*,†^	*P* Value^†^
	Baseline Survey	Completion Survey	Baseline Survey	Completion Survey		
**Knowledge**	**Participants Responding Correctly, N (%)**					
Q1. Infection: contacts	55 (98)	54 (96)	42 (98)	41 (95)	1.3 (0.2-9.8)	.78
Q2. Infection: unhealthy behaviors	20 (36)	20 (36)	21 (49)	19 (44)	0.9 (0.3-2.3)	.81
Q3. Infection: cold conditions	49 (88)	47 (84)	38 (88)	37 (86)	0.9 (0.3-2.8)	.79
Q4. Infection: untreated illness	45 (80)	35 (62)	32 (74)	26 (60)	1.0 (0.4-2.4)	.91
Q5. Influenza vaccine	33 (59)	40 (71)	19 (44)	24 (56)	0.6 (0.2-1.8)	.38
Q6. Hand hygiene	56 (100)	55 (98)	43 (100)	40 (93)	4.1 (0.4-41.1)	.23
Q7. Cough etiquette	44 (79)	46 (82)	28 (65)	35 (81)	0.7 (0.2-2.3)	.56
Q8. Hand cleaners	9 (16)	13 (23)	6 (14)	7 (16)	1.6 (0.5-5.2)	.42
Q9. Work attendance	45 (80)	47 (84)	34 (79)	31 (72)	2.3 (0.8-6.7)	.14
**Beliefs**	**Participants Responding in the Affirmative,^‡^ N (%)**					
Q1. Vaccine effectiveness	43 (77)	49 (88)	29 (67)	26 (60)	**5.6 (1.7-18.5)**	**.003**
Q2. Vaccine eligibility	43 (77)	44 (79)	28 (65)	28 (65)	1.7 (0.5-6.2)	.41
Q3. Influenza prevention	36 (64)	49 (88)	30 (70)	30 (70)	**3.2 (1.1-9.2)**	**.03**
Q4. Influenza illness	44 (79)	47 (84)	38 (88)	37(86)	1.2 (0.3-4.1)	.80
Q5. Vaccine benefits	40 (71)	44 (79)	30 (70)	33 (77)	1.1 (0.3-3.7)	.89
Q6. Vaccine reactions	36 (64)	45 (80)	30 (70)	28 (65)	**4.4 (1.3-15.3)**	**.02**
**Behavior**	**Participants Responding in the Affirmative,^‡^ N (%)**					
Q1.a. Hand hygiene	48 (86)	50 (89)	40 (93)	40 (93)	0.9 (0.2-4.4)	.88
Q1.b. Hand hygiene	37 (66)	47 (84)	28 (65)	35 (81)	1.2 (0.4-3.8)	.75
Q1.c. Hand hygiene	41 (73)	48 (86)	38 (88)	37 (86)	1.9 (0.5-7.6)	.36
Q2.a. Cough etiquette	46 (82)	38 (68)	24 (56)	31 (72)	0.7 (0.3-1.6)	.37
Q2.b. Cough etiquette	37 (66)	52 (93)	28 (65)	37 (86)	2.3 (0.5-9.6)	.27
Q2.c. Cough etiquette	30 (54)	28 (50)	27 (63)	22 (51)	1.0 (0.4-2.5)	.93
Q2.d. Cough etiquette	49 (88)	55 (98)	38 (88)	39 (91)	5.7 (0.6-53.4)	.13
Q2.e. Cough etiquette	31 (55)	44 (79)	23 (53)	30 (70)	1.8 (0.6-5.1)	.30
Q2.f. Cough etiquette	19 (34)	33 (59)	16 (37)	25 (58)	1.1 (0.5-2.7)	.81

^*^Logistic regression model controlling for baseline responses.

^†^Statistically significant effects indicated in bold.

^‡^Refers to responses indicating beliefs or behaviors conducive to preventing influenza illness.

We also examined the rate of influenza immunization among participants during the study period and the rate of work attendance despite a respiratory illness. We did not detect a significant difference in the rate of immunization between the intervention and control groups (24% [13/54] vs 19% [8/43], respectively; *P* = .50). There were a total of 76 participants who reported at least one respiratory illness during the study period, with 21 missing work as a result of an illness. A higher proportion of participants in the intervention group (39%, 16/41) stayed home during an illness compared with participants in the control group (14%, 5/35; *P* = .02).

#### Vaccination Rate Among Household Members

Participants provided information on 160 household members, among which 158 were eligible for the influenza vaccine (two were younger than 6 months at the start of the study and therefore not eligible): 15.8% (13/82) of household members in the intervention group and 9.2% (7/76) in the control group received the influenza vaccine during the study period (*P* = .21).

#### Changes in Knowledge and Behavior in the Control Group

Table 3 shows responses to the survey questions evaluating knowledge and behavior around cardiovascular health and sun protection in the control group. At the end of the study, participants in the control group were more likely to recognize “pain or discomfort in the jaw, neck, or back” and “feeling weak, lightheaded, or faint” as signs of a heart attack and “sudden trouble seeing in one or both eyes” and “severe headache with no known cause” as signs of a stroke. The intervention did not significantly affect the other knowledge elements tested or the proportion of subjects taking medication for their high blood pressure, having their cholesterol checked, or taking measures toward sun protection.

**Table 3:** Changes in knowledge and behavior regarding cardiovascular health and sun protection in the control group (N = 43)

	Baseline Survey	Completion Survey		McNemar Test, *P* Value^*^
**Knowledge**	**Participants Responding Correctly, N (%)**			
Q1.a. Heart attack recognition	18 (42)		34 (79)	**.007**
Q1.b. Heart attack recognition	23 (53)		33 (77)	**.02**
Q1.c. Heart attack recognition	41 (95)		43 (100)	N/A
Q1.d. Heart attack recognition	19 (44)		14 (33)	.16
Q1.e. Heart attack recognition	35 (81)		38 (88)	.18
Q1.f. Heart attack recognition	35 (81)		39 (91)	.20
Q2.a. Stroke recognition	39 (91)		42 (98)	.18
Q2.b. Stroke recognition	40 (93)		43 (100)	N/A
Q2.c. Stroke recognition	33 (77)		39 (91)	**.01**
Q2.d. Stroke recognition	21 (49)		19 (44)	.48
Q2.e. Stroke recognition	37 (86)		41 (95)	.10
Q2.f. Stroke recognition	25 (58)		33 (77)	**.02**
Q3. Interventions	40 (93)		43 (100)	N/A
**Behavior**	**Participants Responding in the Affirmative,^†^ N (%)**			
Q1. High blood pressure	3 (33)		3 (33)	1.0
Q2. Cholesterol monitoring	40 (93)		40 (93)	1.0
Q3.a. Heart disease prevention	33 (77)		32 (74)	.74
Q3.b. Heart disease prevention	34 (79)		33 (77)	.74
Q3.c. Heart disease prevention	34 (79)		31 (72)	.32
Q4.a. Sun protection	20 (47)		22 (51)	.59
Q4.b. Sun protection	28 (65)		32 (74)	.21
Q4.c. Sun protection	25 (58)		26 (60)	.76

^*^Statistically significant effects indicated in bold.

^†^Refers to responses indicating beliefs or behaviors conducive to preventing influenza illness.

### Usability and Utility of the PCHR-Based Program

Of the 123 participants who completed the baseline survey in time to be included in the study, the average number of days to complete the survey was 1.8 days (range 0-25 days) and 1.4 days (range 0-20 days) among intervention and control group participants, respectively. Among the 119 participants who completed at least one biweekly survey, the mean time to completion among intervention subjects (N = 67) was 3.3 days (range 0-24 days) and among control subjects (N = 52), 3.1 days (range 0-15 days). The mean number of completed biweekly surveys was 6.6 (range 1-7) for the intervention group and 6.7 (range 1-7) for the control group. A total of 80% (99/123) of participants completed the exit survey, with mean times to completion of 6.3 days (range 0-27 days) for the intervention group (N = 56) and 7.6 days (range 0-23 days) for the control group (N = 43). Completion rates were 80% (56/70) and 81% (43/53) among the intervention and control groups, respectively.

Among the participants who completed the exit survey, 78% (77/99) rated the PCHR as “extremely” or “very” easy to use and 84% (83/99) indicated survey questions were “extremely” or “very” clear. When asked about specific parts of the messaging system, the aspects deemed most useful by participants were messages with information on prevention of influenza illness, general information on influenza illness, and messages indicating influenza activity in participant's’ geographic area. Overall, 73% (72/99) responded that they would be “extremely” or “very” likely to participate again in the use of a PCHR-based health promotion program such as this one. In terms of privacy concerns for providing information electronically, 57% (56/99) were “not at all” or “a little” concerned, and an additional 25% (25/99) were “moderately” concerned. A total of 62% (61/99) indicated that they would have been willing to provide additional health-related information. Participants found the biweekly surveys to be brief, with 51% (50/99) responding that completion took less than 5 minutes.

Among participants in the intervention group, 54% (30/56) rated the messaging system as “extremely” or “very” useful in providing information about influenza, 13% (7/56) indicated that the messaging system was “extremely” or “very” important in their decision about whether to obtain the influenza vaccine for themselves, and 20% (11/56) responded the same regarding its importance in the immunization of household members.

## Discussion

This study evaluated the use of a PCHR-based program for the promotion of positive health behaviors in a workforce population. With a small sample size, the intervention did not demonstrate a significant effect on the majority of the knowledge, beliefs, and behavior elements tested in either the intervention or the control group. However, the study did demonstrate the feasibility of using a PCHR for health promotion in the workplace, with timely responses from participants, high completion rates, and positive feedback from participants regarding the usability and utility of the PCHR-based program.

The small sample size limits interpretation of our results. This was, in part, due to the timing of corporate restructuring at Hewlett Packard, which occurred during the initial recruitment period and resulted in a reduction in eligible participants as well as a decrease in employee interest in a research trial. A post hoc power calculation reveals that given the number of subjects enrolled, a 28% difference in outcome rates between the two arms would have been required to reject the null hypothesis. The majority of the intergroup and intragroup comparisons trended toward a positive effect but did not reach statistical significance, which may be attributable to low power. Another limitation is the short duration of the trial to assess changes in people’s beliefs and behaviors surrounding health issues. Sustained education and messaging spanning a second influenza season might strengthen the intervention.

One of the strengths of this type of PCHR-based program is that it could be implemented in most work settings in which employees have Internet access. Prior studies have established the feasibility of Web-based health promotion programs with good enrollment and retention rates [16,17], demonstrating employee acceptance of such interventions. There were very few exclusion criteria in our study, and with a Spanish-language version, this type of program would be accessible to most US employees in diverse work settings and geographic areas. The Web-based format gained high acceptance and gave participants flexibility in deciding when to complete the surveys. Our high completion rates are reflective of the convenience and brevity of the intervention, which made it generally appealing to employees.

Another advantage of this type of program is that it can be tailored to a variety of settings and health issues. Potential settings include clinic populations, student bodies, and members of specific organizations such as smoking cessation groups. Issues ranging from nutrition and weight control to binge drinking, safety belt use, and diabetes management could be targeted [18,19]. Within a given setting, appropriate interventions could also be chosen based on the content of the PCHR and the known health issues of the user.

To our knowledge, this is the first study to examine the use of PCHRs as a tool for health promotion in an employee health program. Although the program did not significantly improve the majority of knowledge, beliefs, and behaviors surrounding influenza prevention, the results are promising enough to suggest benefit in a larger follow-up study over a longer period of time.

Overall, this study provides important evidence for the feasibility and utility of using PCHR-based programs for workplace health promotion. There is a growing movement for employers to offer health promotion services in the workplace [20], and several large companies are in the process of implementing PCHRs for their employees [21]. PCHR-based programs provide a flexible, easily accessible option that can be readily adapted to the specific needs of a workforce population or an individual. Further studies are warranted to explore the use of PCHR-based employee health promotion programs and to identify health issues most suitable to this type of program.

## Multimedia Appendix 1

PING screenshots

## Multimedia Appendix 2

Enrollment survey (abbreviated)

## Multimedia Appendix 3

Biweekly survey

## Multimedia Appendix 4

Sample health message

## Multimedia Appendix 5

Control group monthly bulletin

## Abbreviations

ACIP: Advisory Committee on Immunization Practices
CDC: Centers for Disease Control and Prevention
PCHR: personally controlled health record
