# Supplementary material for: Evaluation of Influenza Prevention in the Workplace Using a Personally Controlled Health Record: Randomized Controlled Trial
Source: J Med Internet Res. 2008 Mar 14;10(1):e5. doi: 10.2196/jmir.984 (PMC2483848; doi:10.2196/jmir.984)

Multimedia Appendix

This is a Multimedia Appendix to a full manuscript published in the J Med Internet Res, for full copyright and citation information see http://dx.doi.org/10.2196/jmir.984.

PING Screenshots

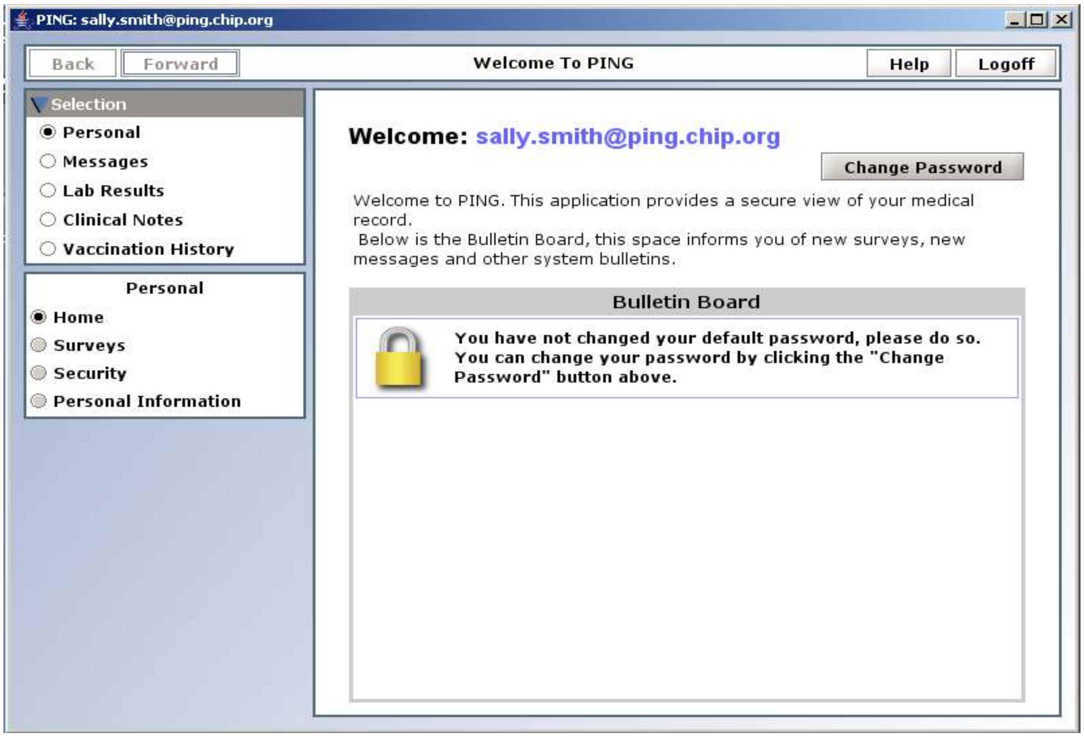

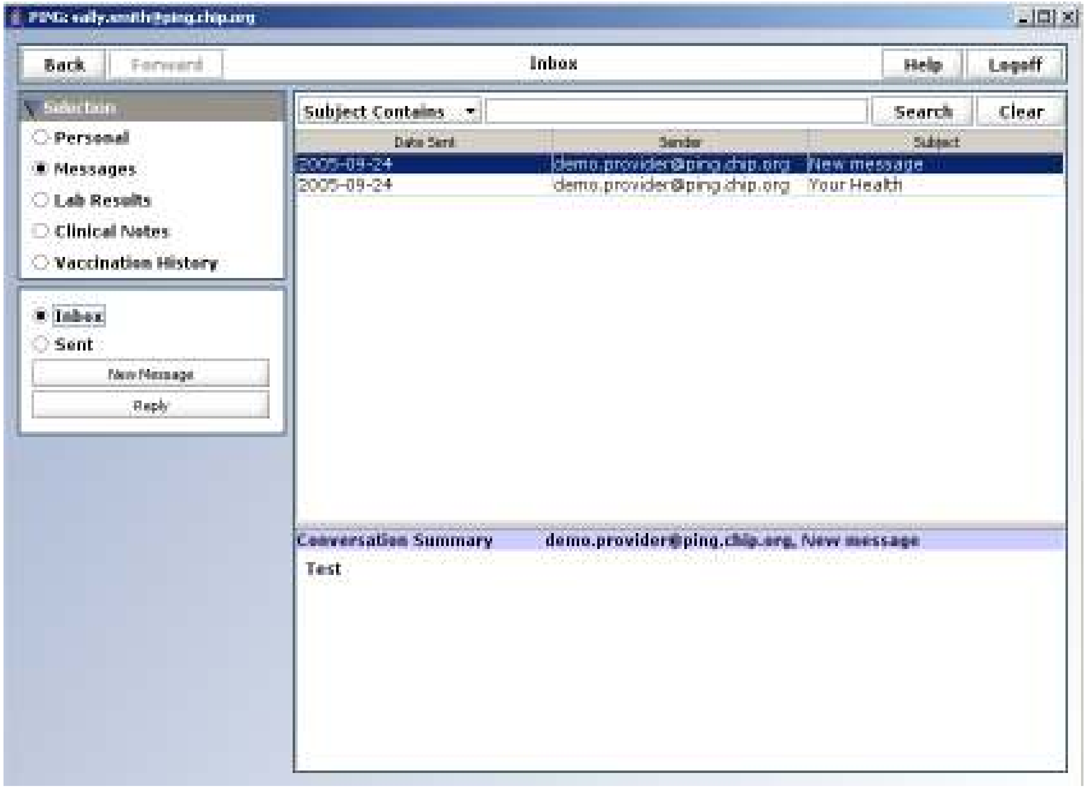

Supplement: Supplementary file 1 [file jmir_v10i1e5_app1.pdf]
