# Supplementary material for: Evaluation of Influenza Prevention in the Workplace Using a Personally Controlled Health Record: Randomized Controlled Trial
Source: J Med Internet Res. 2008 Mar 14;10(1):e5. doi: 10.2196/jmir.984 (PMC2483848; doi:10.2196/jmir.984)
Supplement: Supplementary file 2 [file jmir_v10i1e5_app2.pdf]

Multimedia Appendix

This is a Multimedia Appendix to a full manuscript published in the J Med Internet Res, for full copyright and citation information see http://dx.doi.org/10.2196/jmir.984.

Enrollment Survey (abbreviated)- Intervention arm

Section title: Your address

Please fill in your name and home address:

Name (First Last):_______________________________________

Street name: ____

City: ____

State and Zip: ____

Section title: General information about yourself/your family

1. *Please list all people who live in the same household with you. Fill in their ages and their relationship to you (e.g. son, uncle, live-in nanny). A household member is anyone who spends at least 4 nights in the same home with you.*

	Name	Age, Gender	Relationship
1.	You	________	
2.	___________________________	________	_________________
3.	___________________________	________	_________________
4.	___________________________	________	_________________
5.	___________________________	________	_________________
6.	___________________________	________	_________________
7.	___________________________	________	_________________
8.	___________________________	________	_________________
9.	___________________________	________	_________________
10.	___________________________	________	_________________

2. *For each household member, please indicate whether they work or attend school or daycare. Fill in the number of days per week for work, school, or daycare including half days (e.g. work 3.5 days per week).*

Household Member	Work	Number of days per week	School/Daycare	Number of days per week
1.	☐ Yes<br>☐ No	_______	☐ Yes<br>☐ No	______
2.	☐ Yes<br>☐ No	_______	☐ Yes<br>☐ No	______
3.	☐ Yes<br>☐ No	_______	☐ Yes<br>☐ No	______
4.	☐ Yes<br>☐ No	_______	☐ Yes<br>☐ No	______
5.	☐ Yes<br>☐ No	_______	☐ Yes<br>☐ No	______
6.	☐ Yes<br>☐ No	_______	☐ Yes<br>☐ No	______
7.	☐ Yes<br>☐ No	_______	☐ Yes<br>☐ No	______
8.	☐ Yes<br>☐ No	_______	☐ Yes<br>☐ No	______
9.	☐ Yes<br>☐ No	_______	☐ Yes<br>☐ No	______
10.	☐ Yes<br>☐ No	_______	☐ Yes<br>☐ No	______

3. *Do you have asthma?*

- ☐ yes
- ☐ no
- ☐ not sure

3a. *Have you required regular medical follow up or hospitalizations during the past year for one of the following? Check all that apply.*

- a chronic disorder of the lungs (other than asthma)
- a chronic disorder of the heart
- a chronic metabolic disease (including diabetes mellitus)
- renal dysfunction
- a hemoglobinopathy (including sickle cell anemia, hemoglobin C disease, hemoglobin S-C disease, and thalassemia)
- immune suppression (including immune suppression caused by medications or by an illness)
- any condition that can compromise respiratory function or increase the risk for aspiration (including spinal cord injury, seizure disorder, and neuromuscular disorders)

4. [Ask subject only]
Over the past 3 months, how has your general health been?

- Excellent
- Very good
- Good
- Fair
- Poor

5. [Ask subject only]
For each of the following, have you had this screening in the past 2 years? Choose all that apply.

i. Dental check up (Y/N)
ii. General physical exam (Y/N)
iii. Eye exam (Y/N)

6. [Do not ask for subject]
Does any household member work in a nursing home or other healthcare field and have direct contact with patients?

- Yes, household member number(s): _____ _____ _____ _____
- No

7. *Is any household member pregnant? [ask only for female subjects or household members>14]*

- Yes, household member number(s): _____ _____ _____ _____
- No

8. *Do you now smoke cigarettes every day, some days, or not at all?*

- Everyday
- Some days
- Not at all

9. *Among your household members, who received the flu vaccine during the past flu season (October 2004 through March 2005)?*

Household Member	Received flu vaccine	Household Member	Received flu vaccine
1.	□ Yes<br>□ No<br>□ Not sure	6.	□ Yes<br>□ No<br>□ Not sure
2.	□ Yes<br>□ No<br>□ Not sure	7.	□ Yes<br>□ No<br>□ Not sure
3.	□ Yes<br>□ No<br>□ Not sure	8.	□ Yes<br>□ No<br>□ Not sure
4.	□ Yes<br>□ No<br>□ Not sure	9.	□ Yes<br>□ No<br>□ Not sure
5.	□ Yes<br>□ No<br>□ Not sure	10.	□ Yes<br>□ No<br>□ Not sure

9a. [ask of subjects only if subject answers 'no' to question 9]
Which of the following statements best describes the reason you were not vaccinated?

- □ There was no vaccine available or the physician refused to give me the vaccine because of the shortage when I went to get it
- □ The physician refused to give me the vaccine because of medical reasons
- □ I wanted to save the vaccine for others because of the shortage
- □ The times the vaccine was available were inconvenient
- □ I thought I was not eligible for the vaccine and did not try to get it
- □ I thought the vaccine was not necessary for me
- □ I was concerned that the vaccine does not work
- □ I was worried about potential side effects from the vaccine
- □ I did not have access to a clinic to try to get the vaccine
- □ Other reason

9b. *Among your household members, who received the flu vaccine for the upcoming/current flu season (October 2005 through March 2006)? (same response options as question 9)*

9c. [Ask for subject and every household member if received vaccine for 2005-2006 season]
Where did you/household member get the flu shot?

- ☐ A doctor's office
- ☐ A hospital or emergency room
- ☐ Another type of clinic or health center
- ☐ A senior, recreation, or community center
- ☐ A store
- ☐ Workplace
- ☐ Other

10. [ask of subject only]
Did you get the flu shot during the season before last (October 2003 through March 2004)?

- ☐ Yes
- ☐ No
- ☐ Not sure

10a. [if answer is 'no' or 'not sure' for questions 9 and 10]
Have you ever gotten the flu shot?

- ☐ Yes
- ☐ No
- ☐ Not sure

11. [ask of subject only if subject answers 'yes' to question 9, 10, or 10a]
Have you ever felt sick as a result of the flu shot?

- ☐ Yes
- ☐ No

12. *Compared to other people similar to you, how likely do you think you are to get the flu?*

- ☐ Much less
- ☐ Slightly less
- ☐ About the same
- ☐ Slightly more
- ☐ Much more

13. *If you were to get the flu, how severe do you think it would be compared to other people similar to you?*

- ☐ Much less
- ☐ Slightly less
- ☐ About the same
- ☐ Slightly more
- ☐ Much more

14. *When thinking of everything involved in getting the flu vaccine, how hard or easy would it be to get a flu shot?*
    - Extremely difficult
    - Very difficult
    - Moderately difficult
    - A little difficult
    - Not difficult at all

Section title: What are your opinions on the flu?

1. *Which of the following do you think describes how people get the flu:*
   a. People get influenza through contact with other people (e.g. inhaling germs transmitted by sneezing or coughing, shaking hands, or having close physical contact)
      - Yes
      - No
      - Not sure

   b. People get influenza when because of unhealthy behaviors (e.g. not getting enough rest, not taking vitamins, or not eating healthily)
      - Yes
      - No
      - Not sure

   c. People get influenza because they have been exposed to cold conditions (e.g. not dressing warmly in cold weather or getting wet or chilled)
      - Yes
      - No
      - Not sure

   d. People get influenza when they allow a cold to go untreated, which may result in worsening symptoms and eventually the flu.
      - Yes
      - No
      - Not sure

2. *How effective do you think the flu shot is in reducing your chances of getting the flu or in reducing the severity of the flu if you do get it?*
   - Extremely effective
   - Very effective
   - Moderately effective
   - Slightly effective
   - Not effective at all

3. *Can getting the flu shot give you the flu?*
   - Yes
   - No

4. *Which of the following do you think describes how people can prevent getting or spreading the flu?*
   a. Frequent hand washing is an important way for people to protect themselves from getting the flu
      - Yes
      - No
      - Not sure

   b. To prevent spreading germs, people should sneeze or cough into their upper sleeve if they do not have a tissue available
      - Yes
      - No
      - Not sure

   c. Alcohol-based hand cleaners are not a good way to kill germs on people's hands
      - Yes
      - No
      - Not sure

   d. When people are sick, it is OK for them to go to work as long as they avoid close contact with other people (T/F)
      - Yes
      - No
      - Not sure

5. *Do you think that you should get the flu shot or that it is only for other people?*
   - I should get the flu shot
   - The flu shot is only for other people

6. *Do you agree or disagree with the following statement: There are a number of things I can do to ensure that I do not get the flu.*
   - Disagree strongly
   - Disagree
   - Neither agree nor disagree
   - Agree
   - Agree strongly

7. *How serious of an illness do you think the flu is?*
   - Extremely serious
   - Very serious
   - Moderately serious
   - A little serious
   - Not serious at all

8. *Do you think it is important to get the flu vaccine because it can help keep other people (such as family members) healthy?*
   - Yes
   - No

9. *How likely do you think it is that the flu vaccine would cause a person to have a severe reaction?*
   - Extremely likely
   - Very likely
   - Moderately likely
   - A little likely
   - Not likely at all

Section title: Using the internet

1. *Do you have internet access at home?*
   - Yes
   - No

2. *How comfortable are you using the internet?*
   - Extremely comfortable
   - Very comfortable
   - Moderately comfortable
   - A little comfortable
   - Not comfortable at all

3. *Which best describes your use of the internet?*
   - I use the internet for both work-related and personal tasks
   - I use the internet, but only for work-related tasks
   - I use the internet, but only for personal tasks
   - I rarely use the internet

4. *Which of the following online services, if any, do you use? Please choose* ***all*** *that apply.*
   - I use the internet to read the news
   - I use the internet for banking-related tasks
   - I use the internet to pay bills
   - I shop on the internet
   - I use the internet to get directions
   - I use the internet to download music
   - I use the internet to make travel plans or book tickets
   - I rarely use the internet

5. *How comfortable are you with providing personal information to secure online sites?*
   - ☐ Extremely comfortable
   - ☐ Very comfortable
   - ☐ Moderately comfortable
   - ☐ A little comfortable
   - ☐ Not comfortable at all

6. *How worried are you about privacy invasion for information you give over the internet?*
   - ☐ Extremely worried
   - ☐ Very worried
   - ☐ Moderately worried
   - ☐ A little worried
   - ☐ Not worried at all
