# Supplementary material for: Evaluation of Influenza Prevention in the Workplace Using a Personally Controlled Health Record: Randomized Controlled Trial
Source: J Med Internet Res. 2008 Mar 14;10(1):e5. doi: 10.2196/jmir.984 (PMC2483848; doi:10.2196/jmir.984)
Supplement: Supplementary file 3 [file jmir_v10i1e5_app3.pdf]

Multimedia Appendix

This is a Multimedia Appendix to a full manuscript published in the J Med Internet Res, for full copyright and citation information see http://dx.doi.org/10.2196/jmir.984.

Biweekly survey- Intervention arm

Section title- Recent Illnesses

1. *Since completing the last survey have any household members developed a NEW respiratory illness, such as the flu, a cold, a sinus infection, or bronchitis? If so, what symptoms have they had? Please check the appropriate boxes.*

If no one has been sick with the flu, a cold, or other respiratory infection, skip to ***Section 2****.*

Household Member	Illness	Symptoms	
1. You	☐ Yes<br>☐ No	☐ fever<br>☐ cough<br>☐ sore throat<br>☐ muscle aches<br>☐ headache	☐ runny nose<br>☐ stuffy nose<br>☐ fatigue<br>☐ breathing difficulty<br>☐ voice hoarseness
2. _____________	☐ Yes<br>☐ No	☐ fever<br>☐ cough<br>☐ sore throat<br>☐ muscle aches<br>☐ headache	☐ runny nose<br>☐ stuffy nose<br>☐ fatigue<br>☐ breathing difficulty<br>☐ voice hoarseness
3. _____________	☐ Yes<br>☐ No	☐ fever<br>☐ cough<br>☐ sore throat<br>☐ muscle aches<br>☐ headache	☐ runny nose<br>☐ stuffy nose<br>☐ fatigue<br>☐ breathing difficulty<br>☐ voice hoarseness
4. _____________	☐ Yes<br>☐ No	☐ fever<br>☐ cough<br>☐ sore throat<br>☐ muscle aches<br>☐ headache	☐ runny nose<br>☐ stuffy nose<br>☐ fatigue<br>☐ breathing difficulty<br>☐ voice hoarseness
5. _____________	☐ Yes<br>☐ No	☐ fever<br>☐ cough<br>☐ sore throat<br>☐ muscle aches<br>☐ headache	☐ runny nose<br>☐ stuffy nose<br>☐ fatigue<br>☐ breathing difficulty<br>☐ voice hoarseness

2. *Did any of the household members who were sick with a respiratory illness take medications? If yes, (1) were any of them prescription medications, and (2) which medications did they take? Please check **all** that apply.*

Household Member	Med?	Rx?	Medications used
1.	☐ Yes<br>☐ No	☐ Yes<br>☐ No	☐ Tylenol or Ibuprofen (includes Motrin, Advil, and Aleve)<br>☐ over the counter cough or cold medication<br>☐ antibiotic<br>☐ anti-influenza agent<br>☐ other:_______________
2.	☐ Yes<br>☐ No	☐ Yes<br>☐ No	☐ Tylenol or Ibuprofen (includes Motrin, Advil, and Aleve)<br>☐ over the counter cough or cold medication<br>☐ antibiotic<br>☐ anti-influenza agent<br>☐ other:_______________
3.	☐ Yes<br>☐ No	☐ Yes<br>☐ No	☐ Tylenol or Ibuprofen (includes Motrin, Advil, and Aleve)<br>☐ over the counter cough or cold medication<br>☐ antibiotic<br>☐ anti-influenza agent<br>☐ other:_______________
4.	☐ Yes<br>☐ No	☐ Yes<br>☐ No	☐ Tylenol or Ibuprofen (includes Motrin, Advil, and Aleve)<br>☐ over the counter cough or cold medication<br>☐ antibiotic<br>☐ anti-influenza agent<br>☐ other:_______________
5.	☐ Yes<br>☐ No	☐ Yes<br>☐ No	☐ Tylenol or Ibuprofen (includes Motrin, Advil, and Aleve)<br>☐ over the counter cough or cold medication<br>☐ antibiotic<br>☐ anti-influenza agent<br>☐ other:_______________

3. *Did they have to see or speak with a healthcare provider because of the respiratory illness?*

Household Member		Healthcare use
1.	☐ Yes<br>☐ No	☐ saw doctor in primary care clinic<br>☐ called doctor or nurse<br>☐ seen in the ER<br>☐ admitted to hospital<br>☐ saw doctor in specialty clinic<br>☐ other:_____________
2.	☐ Yes<br>☐ No	☐ saw doctor in primary care clinic<br>☐ called doctor or nurse<br>☐ seen in the ER<br>☐ admitted to hospital<br>☐ saw doctor in specialty clinic<br>☐ other:_____________
3.	☐ Yes<br>☐ No	☐ saw doctor in primary care clinic<br>☐ called doctor or nurse<br>☐ seen in the ER<br>☐ admitted to hospital<br>☐ saw doctor in specialty clinic<br>☐ other:_____________
4.	☐ Yes<br>☐ No	☐ saw primary care doctor in clinic<br>☐ called doctor or nurse<br>☐ seen in the ER<br>☐ admitted to hospital<br>☐ saw doctor in specialty clinic<br>☐ other:_____________
5.	☐ Yes<br>☐ No	☐ saw primary care doctor in clinic<br>☐ called doctor or nurse<br>☐ seen in the ER<br>☐ admitted to hospital<br>☐ saw doctor in specialty clinic<br>☐ other:_____________

3a. [If yes for question 3; ask for each household member who saw a physician]
Did you/household member get a flu test?

☐ Yes
☐ No

3b. [If yes to question 1a; ask for each household member who got the test]
What was the result of the test?

☐ positive for the flu
☐ negative for the flu
☐ don't know

4. [Ask for every household member who was sick]
For how many days did the respiratory illness last from beginning to end?

Number of days: ☐ don't know yet, the illness is still present
☐ 1, 2, 3, 4, etc to 14

5. *Did any of the household members who were sick with a respiratory illness have to miss work, school, or daycare because they were sick? Did any household members have to miss work or school to take care of another household member sick with a respiratory illness? Please check the appropriate boxes.*

Household Member	Missed Due to Illness	Missed Due to Sick family member
1.	☐ work; # of days:______	☐ work; # of days:______
2.	☐ work; # of days:______<br>☐ school; # of days:______<br>☐ daycare; # of days:______	☐ work; # of days:______<br>☐ school; # of days:______
3.	☐ work; # of days:______<br>☐ school; # of days:______<br>☐ daycare; # of days:______	☐ work; # of days:______<br>☐ school; # of days:______
4.	☐ work; # of days:______<br>☐ school; # of days:______<br>☐ daycare; # of days:______	☐ work; # of days:______<br>☐ school; # of days:______
5.	☐ work; # of days:______<br>☐ school; # of days:______<br>☐ daycare; # of days:______	☐ work; # of days:______<br>☐ school; # of days:______

6. [Ask for subject only]
How many days did you attend work despite feeling sick from the respiratory illness?
Number: ______________________

7. *During the past 2 weeks have you been sick with an illness other than a respiratory illness?*
☐ Yes
☐ No

If so, did you miss any days of work because of this illness?
☐ Yes; # of days: _______________
☐ No

Section title- Flu shot

1. [Ask for household members who have not yet gotten the flu shot, including the subject] *Since completing the last survey, have you/ household members received the flu shot?*

 Yes. Household member number(s): _____ _____ _____ _____ _____
 No

2. [Ask for subject and every household member if received vaccine]
Where did you/household member get the flu shot?

 A doctor's office or health maintenance organization
 A health department
 Another type of clinic or health center
 A senior, recreation, or community center
 A store
 A hospital or emergency room
 Workplace
