# Supplementary material for: Evaluation of Influenza Prevention in the Workplace Using a Personally Controlled Health Record: Randomized Controlled Trial
Source: J Med Internet Res. 2008 Mar 14;10(1):e5. doi: 10.2196/jmir.984 (PMC2483848; doi:10.2196/jmir.984)
Supplement: Supplementary file 4 [file jmir_v10i1e5_app4.pdf]

Multimedia Appendix

This is a Multimedia Appendix to a full manuscript published in the J Med Internet Res, for full copyright and citation information see http://dx.doi.org/10.2196/jmir.984.

Sample Health Message: Household member with respiratory illness

---

According to the information you provided, **Alice is or has recently been sick with a cold or other respiratory illness**. A respiratory illness can be caused by the flu, although it is impossible to tell for sure without getting a flu test.

The flu is different from a cold. It usually comes on suddenly and may include these symptoms:

- Fever
- Headache
- Tiredness (can be extreme)
- Dry cough
- Sore throat
- Runny or stuffy nose
- Body aches

If Alice is still sick, and has these symptoms of the flu, she should **contact her physician** to inquire whether antiviral medications are appropriate for her. She should also ask about when she can receive the flu vaccine since the vaccine may prevent her from getting sick again.

It is also important to take steps to **prevent spreading the flu to others**. Flu viruses are spread by coughing or sneezing. They usually spread from person to person, although sometimes people become infected by touching something with flu viruses on it and then touching their eyes, nose, or mouth.

Here are some ways to help prevent spreading the flu to others:

- ***Avoid close contact.***
  Avoid close contact, such as shaking hands, with people who are sick with respiratory symptoms. When you are sick, keep your distance from others to protect them from getting sick too.

- ***Stay home when you are sick.***
  If possible, stay home from work, school, and errands during the first days when you are sick. You will help prevent others from catching your illness.

- ***Cover your mouth and nose.***
  Cover your mouth and nose with a tissue when coughing or sneezing. It may prevent those around you from getting sick. If you do not have a tissue, cough or sneeze into your upper sleeve, not your hands.

- ***Wash your hands.***
  Washing your hands often with soap and water or an alcohol-based cleaner will help protect you from germs.

- ***Avoid touching your eyes, nose or mouth.***
  Germs are often spread when a person touches something that is contaminated with germs and then touches his or her eyes, nose, or mouth.

The single best way to protect against the flu is to GET THE FLU VACCINE!   According to the information you have provided, **Alice and Bob still need to get the flu vaccine**. It is especially important that **Bob** gets the vaccine since he is **at high risk for serious complications from the flu.**

For more information on the flu vaccine, go to www.cdc.gov/flu/protect/keyfacts.htm.
