# Supplementary material for: Evaluation of Influenza Prevention in the Workplace Using a Personally Controlled Health Record: Randomized Controlled Trial
Source: J Med Internet Res. 2008 Mar 14;10(1):e5. doi: 10.2196/jmir.984 (PMC2483848; doi:10.2196/jmir.984)
Supplement: Supplementary file 5 [file jmir_v10i1e5_app5.pdf]

Multimedia Appendix

This is a Multimedia Appendix to a full manuscript published in the J Med Internet Res, for full copyright and citation information see http://dx.doi.org/10.2196/jmir.984.

Control Group Monthly Bulletin: Recognition of heart attacks

Coronary heart disease is America's #1 killer. By knowing to recognize the signs of a heart attack and understanding the importance of calling 9-1-1 immediately, ***YOU can help reduce the mortality of heart attacks.***

Did you know?

- Each year, cardiovascular disease and sudden cardiac arrest claim the lives of 335,000 Americans before they reach a hospital.
- Almost 80 percent of cardiac arrests occur at home and are witnessed by a family member.

Do you know how to recognize a heart attack?
Some heart attacks are sudden and intense — the "movie heart attack," where no one doubts what's happening. But most heart attacks start slowly, with mild pain or discomfort. Often people affected aren't sure what's wrong and wait too long before getting help. Here are signs that can mean a heart attack is happening:

- **Chest discomfort.** Most heart attacks involve discomfort in the center of the chest that lasts more than a few minutes, or that goes away and comes back. It can feel like uncomfortable pressure, squeezing, fullness or pain.
- **Discomfort in other areas of the upper body.** Symptoms can include pain or discomfort in one or both arms, the back, neck, jaw or stomach.
- **Shortness of breath.** May occur with or without chest discomfort.
- **Other signs:** These may include breaking out in a cold sweat, nausea or lightheadedness

Dial 9-1-1 fast!
If you or someone you're with has chest discomfort, especially with one or more of the other signs, don't wait longer than a few minutes (no more than 5) before calling for help. **Call 9-1-1... Get to a hospital right away.**

Calling 9-1-1 is almost always the fastest way to get lifesaving treatment. Emergency medical services staff can begin treatment when they arrive -- up to an hour sooner than if someone gets to the hospital by car. The staff are also trained to revive someone whose heart has stopped. Patients with chest pain who arrive by ambulance usually receive faster treatment at the hospital, too.

If you can't access the emergency medical services (EMS), have someone drive you to the hospital right away. If you're the one having symptoms, don't drive yourself, unless you have absolutely no other option.
